# The impact of a graphic novel on anxiety and stress in patients undergoing endoscopic ultrasound with fine needle biopsy for pancreatic lesions: a pilot study protocol

**DOI:** 10.3389/fgstr.2024.1359002

**Published:** 2024-04-02

**Authors:** Giacomo Emanuele Maria Rizzo, Mario Traina, Ilaria Tarantino

**Affiliations:** ^1^ Endoscopy Service, Department of Diagnostic and Therapeutic Services, Institute of the Mediterranean for Transplantation and High Specialty Therapies (ISMETT), Istituto di Ricovero e Cura a Carattere Scientifico (IRCCS), Palermo, Italy; ^2^ Department of Precision Medicine in Medical, Surgical and Critical Care (Me.Pre.C.C.), University of Palermo, Palermo, Italy

**Keywords:** EUS, FNB, graphic novel, pancreas, health care, patient care

## Abstract

The utilization of graphic novels in the realm of clinical medicine is an infrequent occurrence. However, there is a burgeoning interest in their application across a spectrum of pathological conditions with the ultimate aim of enhancing patient care. This study is a prospective pilot designed to assess the influence of graphic novels on the stress levels and behavioral responses of patients diagnosed with pancreatic lesions and who are to undergo endoscopic ultrasound-guided fine needle biopsy (EUS-FNB). Patients exhibiting radiological and clinical pancreatic lesions needing biopsy will be evaluated consecutively. The inclusion criteria encompass the presence of a solid pancreatic mass or a partially solid mass in the event of a cystic component. The exclusion criteria include patients with cognitive impairments, those currently on benzodiazepines or other psychotropic medications, and those with a prior diagnosis of cancer. The authors have developed a comic panel comprising a sequence of six vibrant vignettes, which delineate the standard procedure of EUS-FNB to the patient. Following hospital admission, patients who meet the enrolment criteria and consent to participate in the study will be randomly assigned to either the test or the control group. A graphic novel will be distributed to all patients in the test group, who will have the opportunity to peruse it while awaiting the procedure. Subsequent to the EUS-FNB, all enrolled patients will complete the Beck Anxiety Inventory (BAI) and a modified version of the Depression Anxiety Stress Scales-21 (termed mDASS-21 or mASS-14). The BAI, a 21-item self-report inventory, is employed to gauge the severity of anxiety in adults. The other questionnaire is a modified rendition of the DASS-21, which originally comprised 21 items segregated into three subscales (anxiety, stress, and depression) with seven items each. The anxiety subscale measures physiological arousal, situational anxiety, and the subjective experience of the effects of anxiety, while the stress subscale assesses chronic non-specific arousal, difficulty relaxing, nervous tension, irritability, agitation, and impatience.

## Introduction

Endoscopy, specifically endoscopic ultrasound (EUS), alongside laparoscopic surgery, is the principal modality for the diagnosis and treatment of severe and occasionally life-threatening pancreatic disorders. Particularly in the context of pancreatic lesions, diagnostic procedures such as EUS with fine needle biopsy (EUS-FNB) can induce states of fear and anxiety in patients. The apprehension and/or anxiety associated with the biopsy procedure may be disproportionate relative to the actual risks or objective threat (1). Waiting for a biopsy can be fraught with fear (“What if it is cancer?”), given that a cancer diagnosis is arguably one of the most stressful, life-altering events an individual can encounter (2). The term graphic medicine was coined by a consortium of researchers, clinicians, and artists to describe a new subfield of research and practice (3). It can be defined as a form of visual storytelling exploring narratives in healthcare, cancer, healing, and disability (4). Graphic medicine is currently a trending topic in health communication, with messages contained within the storytelling of health communication being increasingly described as “creative” and “compelling” (5). Researchers are also demonstrating growing interest in graphic narratives, which continue to emerge as efficacious communication tools in public health (6, 7). Various authors theorize the role of storytelling with comics in shaping behaviors, as in the case of anxiety reduction. Graphic medicine is considered effective by many practitioners because it provides medical professionals with an innovative approach to information dissemination, which is both rapid and comprehensive (8). Graphic medicine uses sequential (though not always narratively linear) visual storytelling to convey health-related experiences or information (9). The use of comics facilitates soft skills, thereby opening up a space in which care professionals can communicate effectively with patients while they are going through emotionally charged situations. With their combination of images, limited text, and relatively short format, comics provide an easy option for the effective communication of health-related messages (10). Consequently, graphic novels have been applied to several fields, including oncology (11), HIV infection (12), eating disorders (13), and oral cancer (14). To date, there have been no applications in the field of endoscopy, so the aim of the present study is to evaluate, for the first time in the literature, the efficacy of colorful graphic novels in reducing anxiety in adult patients waiting for an endoscopic-guided biopsy for pancreatic lesions.

## Materials and methods

### Study design

**Figure 1:**
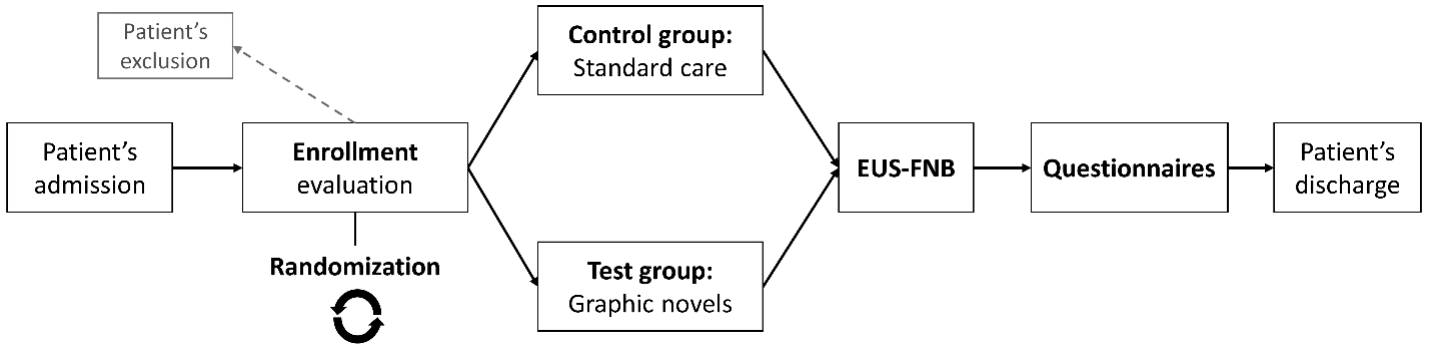
Overview of the study protocol. EUS-FNB, endoscopic ultrasound with fine needle biopsy.

This is a prospective pilot study evaluating the influence of graphic novels on the stress levels and behavioral responses of patients diagnosed with pancreatic lesions and who are to undergo EUS-FNB. The study is being conducted at ISMETT (Istituto Mediterraneo per i Trapianti e Terapie ad Alta Specializzazione), IRCCS (Istituto di Ricovero e Cura a Carattere Scientifico) or the Scientific Institute of Hospitalization and Care in collaboration with UPMC Italy (University of Pittsburgh Medical Center, Italy), located in Palermo, Italy. The study adheres to the principles of the Declaration of Helsinki regarding experimentation involving human subjects, and written informed consent will be procured from all enrolled participants. The protocol is under evaluation of the ethical committee with number IRRB/43/23 and it is registered to ClinicalTrials.gov (NCT06268106). The authors will consecutively evaluate all patients with radiological and clinical suspicion of a pancreatic lesion from from May 2024 to October 2024. The inclusion criteria are (1) age ≥ 18 years; (2) pancreatic solid mass or partially solid in the case of a cystic component requiring tissue acquisition; (3) agreement to participate in the study by signing the informed consent form, and the ability to read and understand the informed consent form; and (4) patients not affected by any known psychological disorder. The exclusion criteria are (1) patients with cognitive deficits, such that they cannot adequately complete the questionnaire, and the visually impaired; (2) suspected or obvious pregnancy status in female patients; (3) patients using benzodiazepines or other psychotropic medications; and (4) patients with a previous diagnosis of cancer. Following hospital admission, patients successfully enrolled and consenting to participate in the study will be randomly allocated to the test or control group using dedicated software (Random Allocation Software, version 1.0) (15). Patients randomized to the control group will experience the standard daily routine in case of EUS-FNB of a pancreatic lesion at our institute, including procedure explanation and consent acquisition. Moreover, it will include an initial colloquium with physical examination, during which the physician explains how the day will unfold, addressing any procedure-related and medical care-related questions the patient may have. After the initial explanation of the daily medical routine, the physician will ask the patient to read the informed consent and to sign it, if they agree. The patient then waits in their room until it is their turn to be brought to the endoscopic room for the EUS-FNB. In case of randomization to the test group (**Figure 1**), the graphic novel will be provided to the patients after the initial colloquium, leaving them waiting for the procedure with the graphic novel, allowing them to read it while waiting and to assimilate slower how their day will unfold.

### Graphic novel or comic panel

**Figure 2:**
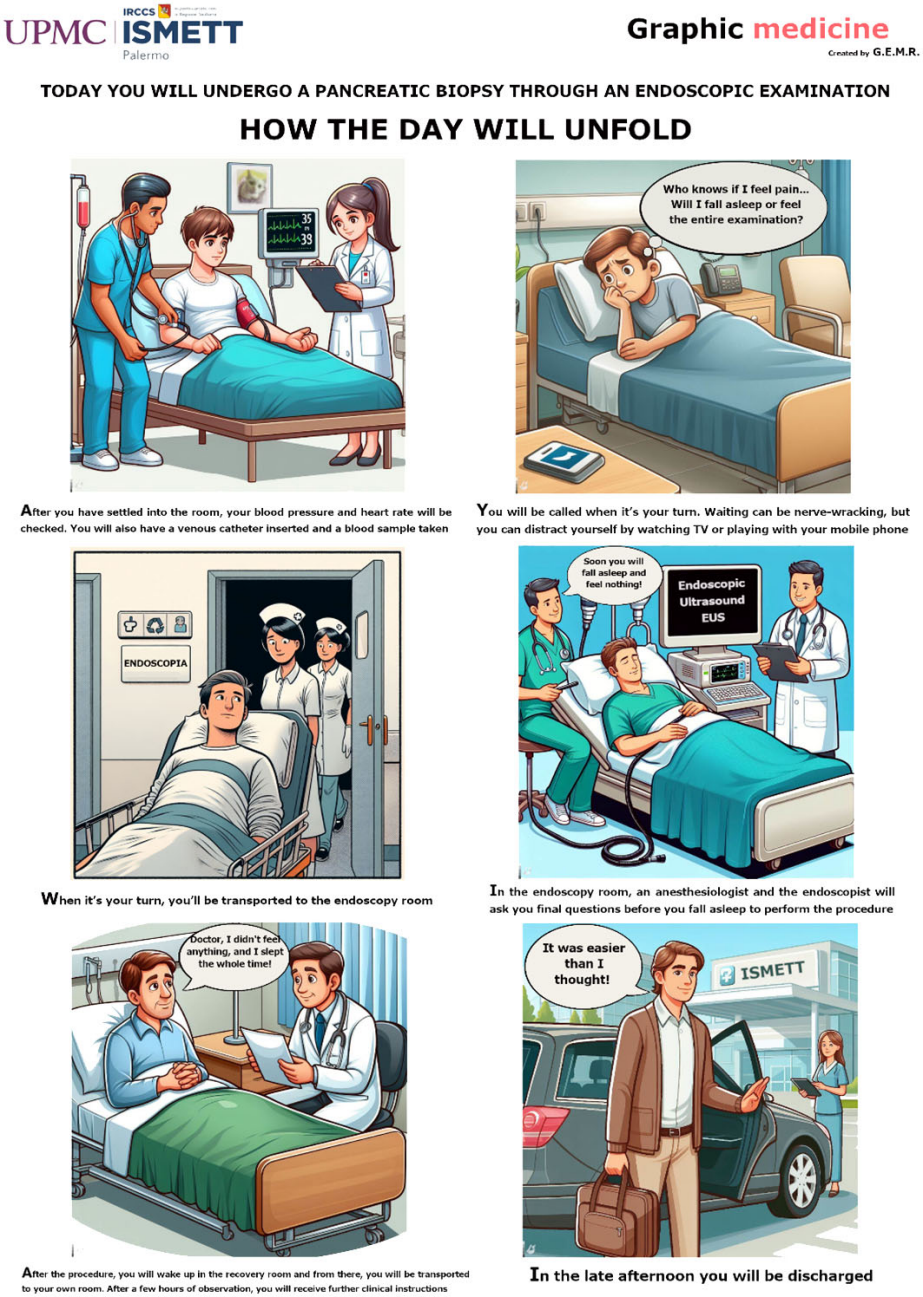
The graphic novel created to administer to patients randomized to the test group.

The authors created a comic panel consisting of a sequence of six colorful vignettes in which the routine procedure of an EUS-FNB is described to the patient (**Figure 2**). The authors focused on various graphical aspects of comics, such as the facial expressions of the characters and the background, both made to look similar to the effective contexts experienced by patients at the institute. The initial drafts of the six vignettes were created using artificial intelligence (AI image creator using DALL-E3 technology) and later modified by one author (G.E.M.R.) using graphical software (GIMP, GNU Image Manipulation Program, version 2.10.28, gimp.org).

### Assessing anxiety

**Figure 3:**
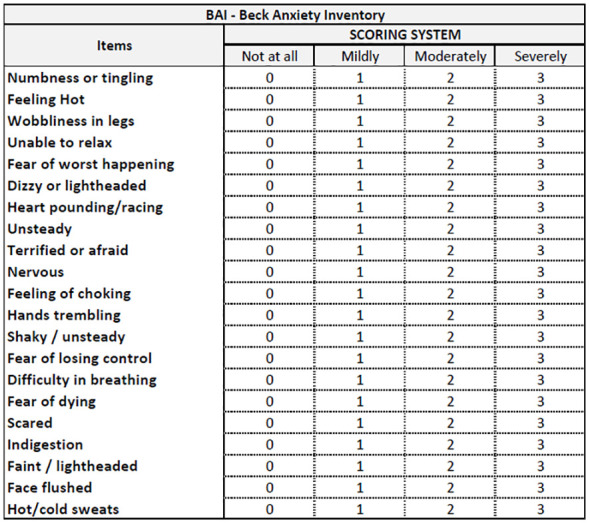
Beck Anxiety Inventory (BAI), from Beck et al. (16).

**Figure 4:**
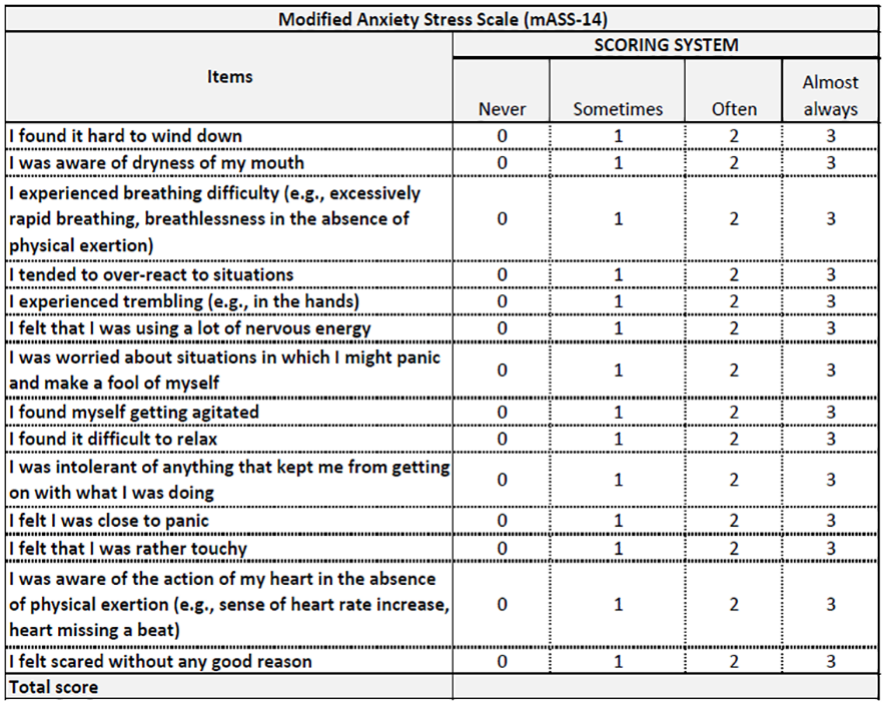
The modified version of DASS-21, named mASS-14.

After EUS-FNB, all the recruited patients will receive the Beck Anxiety Inventory (BAI) (16) and a modified version of the Depression Anxiety Stress Scales-21 (mDASS-21) (17) (**Figure 1**). Prior to the endoscopic procedures (i.e., EUS-FNB), these questionnaires will be administered anonymously in a mixed paper-pencil and/or QR code mode to be used with a personal mobile phone. A physician, nurse or clinical assistant will be present for questions and clarification. The BAI is a 21-question self-report inventory used to assess anxiety severity in adults older than 17 years. Respondents answer questions about common anxiety symptoms, such as numbness, tingling, sweating unrelated to heat, and fear of catastrophic events. The BAI takes approximately 5 to 10 min to complete. Scores range from 0 (not at all) to 3 (severe), with higher scores indicating more severe anxiety. Standardized cutoffs classify anxiety levels as minimal, mild, moderate, or severe. The other questionnaire is a modified version of the DASS-21, which originally consisted of 21 items divided into three subscales (anxiety, stress, and depression) with seven items each. We focused on the anxiety and stress subscales, so we modified the questionnaire by removing the depression subscale. Specifically, the anxiety subscale assesses physiological arousal, situational anxiety, and subjective experience of the effects of anxiety, while the stress subscale evaluates chronic non-specific arousal, difficulty in relaxing, nervous tension, irritability, agitation, impatience, and overactivity. Each item is rated on a 4-point scale from 0 (did not apply to me at all) to 3 (applied to me very much, or most of the time). The total score for each subscale ranges from 0 to 14 in our modified version, with higher scores indicating higher levels of anxiety and stress. We named this modified version mDASS-21 or mASS-14. The mASS-14 has four cutoff points to classify anxiety and stress levels: normal (0–4), mild (5–8), moderate (9–11), or severe (12–14). Since the DASS-21 is a reliable and valid measure of anxiety and stress symptoms with good internal consistency, test–retest reliability, and discriminant validity, we will evaluate its modified version in our scenario. Both the BAI (**Figure 3**) and mDASS-21 (**Figure 4**) used in this pilot study were in the Italian versions to avoid language barriers.

### Data collection

Data will be collected prospectively in a dedicated electronic case report form (eCRF) through a secure platform such as RedCap, and will include gender, age, level of education (years or diploma/degree), marital status (married/cohabiting/single), and children/grandchildren. Medical history, including previous clinically relevant conditions, such as hospitalizations, surgeries, chronic medical therapies, or familiarity with cancer, will also be collected. Data regarding clinical presentation and lesion characteristics will be collected as well. The scale for intervention evaluation will be an Italian version of the Beck Anxiety Inventory (BAI), DASS-21, Cancer Worry Scale (CWS), STAI-S (State-Trait Anxiety Inventory form) and the APAIS (Amsterdam Preoperative Anxiety and Information Scale). ECRFs in RedCap will be fully anonymized using an identification (ID) code and age instead of date of birth. ECRFs will be translated into an electronic spreadsheet for analysis.

### Statistical analysis

The analysis will be performed using jamovi software (the jamovi project, version 2.3, 2023) with R language (R Core Team, 2022). Descriptive statistics will be used to analyze the patients’ socio-demographic and physical characteristics. BAI and mASS-14 scores will be summarized through mean value, standard deviation (SD), maximum, median, and interquartile range (IQR) as necessary. Comparisons between groups will be performed according to the most appropriate statistics (Fisher or χ-squared for categorical variables, parametric or non-parametric statistics for comparisons of continuous variables). Any relation between BAI and mASS-14 scores will be measured through a linear correlation coefficient.

## Discussion

The utilization of graphic narratives in the medical field, though not widespread, is gaining recognition for its potential in patient care management across various pathological conditions. The American Cancer Society has pioneered this approach in cancer prevention, employing a comic book to address the barriers to cervical cancer screening. This innovative method bridges contemporary research with historical public health communication efforts, highlighting the promising attributes of this tool (11). In the sensitive domain of acquired immunodeficiency syndrome (AIDS), comic books have contributed positively to the discourse surrounding this infectious and severe condition (12). Therefore, graphic narratives have been demonstrated to encourage true education, understanding, and empathy. Another significant role of graphic novels is their impact on stress and anxiety management in patients awaiting biopsy for potentially malignant disorders. In a recent open-label randomized clinical trial, graphic novels significantly improved the ability of patients to tolerate anxiety while waiting for an oral biopsy in suspected oral cancers (14). Nowadays, feelings of anxiety and stress are pervasive, which can increase in the case of doubtful health conditions needing medical procedures, such as a pancreatic lesion biopsy. Patients can experience intense and immediate fear and/or anxiety prior to an endoscopic treatment and/or pancreatic biopsy, and this discomfort typically manifests in the waiting room, possibly leading to negative intrusive thoughts (e.g., “It will hurt.” or “I will be diagnosed with a terrible disease.”) (18). Graphic novels are emerging as valuable medical tools to support patient care, and comics can easily communicate concepts by telling stories with relevant characters, settings, and situations. Our pilot study aims to give novel data about the impact of this tool in clinical practice for improving clinical psychological patient conditions associated with diagnostic procedures. In the near future, the results of this pilot study could guide physicians and companies in designing new clinical trials to evaluate graphic novels in clinical practice in the management of anxiety and stress tolerance.

## Ethics statement

The studies involving humans were approved by the Ethical CommitteeISMETT, Palermo, Italy. The studies were conducted in accordance with the local legislation and institutional requirements. The participants provided their written informed consent to participate in this study.

## Author contributions

GR: Conceptualization, Data curation, Methodology, Resources, Software, Validation, Writing – original draft, Writing – review & editing. MT: Funding acquisition, Supervision, Validation, Writing – review & editing. IT: Funding acquisition, Supervision, Validation, Writing – review & editing.
